# Gibt es ein Post-COVID-Syndrom?

**DOI:** 10.1007/s10405-020-00347-0

**Published:** 2020-10-08

**Authors:** Bernd Lamprecht

**Affiliations:** Klinik für Lungenheilkunde, Kepler Universitätsklinikum GmbH, Krankenhausstr. 9, 4020 Linz, Österreich

**Keywords:** Coronavirus, Fatigue, Funktionelle Einschränkungen, Rehabilitation, Folgeerscheinungen, Coronavirus, Fatigue, Functional restrictions, Rehabilitation, Sequelae

## Abstract

Für kritisch kranke COVID-19-Patienten könnte das Überleben der Akutphase evtl. nur die Bewältigung der ersten Etappe eines insgesamt langen und herausfordernden Weges sein. Körperliche, kognitive und psychologische Folgen sind realistisch. Aber stellen residuale Symptome bei Patienten mit mikrobiologischer Normalisierung tatsächlich ein „Post-COVID-Syndrom“ dar, und welche Symptome sind in diesem Zusammenhang prinzipiell denkbar und in der Lage, dieses zu begründen? Dass kritisch kranke Patienten oftmals über einen längeren Zeitraum nach ihrer Krankenhausentlassung noch funktionelle Einschränkungen erleben, ist nicht neu. Für die Diagnose eines Post-COVID-Syndroms ist es aber in den meisten Fällen bei COVID-19 jetzt noch zu früh. Dafür müssen die Symptome mindestens 6 Monate anhalten. Aktuell kann man daher wohl nur von postinfektiöser Fatigue sprechen. Und selbst wenn sich Betroffene körperlich wieder erholen, so sind sie evtl. besonders gefährdet, an lang anhaltenden mentalen Gesundheitsproblemen zu leiden bzw. eine reduzierte Lebensqualität zu empfinden. Solche Beobachtungen gibt es jedoch nicht nur nach einem ARDS („acute respiratory distress syndrome“), viele Intensivpatienten verzeichnen lange anhaltende Beschwerden, die auch als „post-intensive care syndrome“ (PICS) bezeichnet werden. In Summe bestehen jedenfalls ausreichend Hinweise für die mögliche Existenz eines „Post-COVID-Syndroms“ bzw. für die Berechtigung, die denkbaren Folgeerscheinungen mit persistierenden Symptomen so zu bezeichnen. Es sind alle Anstrengungen gerechtfertigt, die eine vollständige funktionelle Wiederherstellung und eine Rückkehr in ein Leben nach Corona ermöglichen.

## Abstract (en)

For critically ill COVID-19 patients surviving the acute phase of the disease could possibly only mean having overcome the first stage of a long and challenging path. Physical, cognitive and psychological consequences seem to be realistic; however, do residual symptoms in patients who have returned to microbiological normalization constitute a post-COVID syndrome and which symptoms are principally possible in this context and are able to cause such a syndrome? It is no novelty that critically ill patients often still sustain functional limitations over a long period after discharge from hospital, in many cases even over many years. In most cases of COVID-19 it is too early for the diagnosis of a post-COVID syndrome. For this the symptoms would have to have lasted over a period of at least 6 months; therefore, only a post-infection fatigue can currently be spoken of. On top of this, even if patients recover physically they could be at particular risk of suffering from long-term mental health problems or perceive a reduced quality of life. Such findings exist not only after ARDS as many intensive care unit patients sustain long-term disorders, which is also known as post-intensive care syndrome (PICS). To sum up, there is sufficient evidence for the possible existence of a post-COVID syndrome or for the justification to correspondingly designate these possible sequelae with persisting symptoms in this way. In any case, all efforts that enable a complete functional recovery and a return to a life after corona are justified.

Für kritisch kranke COVID-19-Patienten könnte das Überleben der Akutphase evtl. nur die Bewältigung der ersten Etappe eines insgesamt langen und herausfordernden Weges sein. Körperliche, kognitive und psychologische Folgen sind realistisch, zumal dies bei SARS‑1 und auch ARDS („acute respiratory distress syndrome“) in anderem Zusammenhang wiederholt beschrieben wurde. Gerade schwere Verläufe mit komplizierten Intensivaufenthalten und längeren Zeiten mechanischer Beatmung sind ein plausibler Risikofaktor für Folgeerscheinungen bzw. persistierende Symptome. Aber stellen residuale Symptome bei Patienten mit mikrobiologischer Normalisierung tatsächlich ein „Post-COVID-Syndrom“ dar?

Unter dem aus dem Griechischen kommenden Begriff „Syndrom“ verstehen wir in der Medizin das Zusammentreffen bzw. gemeinsame Bestehen mehrerer Symptome. Dabei stellt der primär deskriptive Begriff eine gemeinsame Pathogenese der zugleich vorhandenen Symptome in den Vordergrund. Ein „Post-COVID-Syndrom“ würde also 2 Umstände bedingen:

- einerseits das weitere Bestehen mehrerer Symptome nach Abklingen der akuten Krankheitsphase und
- andererseits deren gemeinsame Ursache in der ursprünglichen SARS-CoV-2-Infektion.

Hier gilt es vorerst, folgende Frage zu beantworten: Welche Symptome sind in diesem Zusammenhang prinzipiell denkbar und in der Lage, ein Post-COVID-Syndrom zu begründen? Die Persistenz von Fatigue, Dyspnoe und neuropsychologischen Symptomen wird in Abhängigkeit der jeweiligen Studienpopulation sehr häufig berichtet, in 35 % bei ambulant behandelten COVID-Patienten [1] und in 87 % bei Hospitalisierten [2].

Dass kritisch kranke Patienten oftmals über einen längeren Zeitraum nach ihrer Krankenhausentlassung noch funktionelle Einschränkungen erleben, in vielen Fällen sogar über die Dauer von mehreren Jahren, ist nicht neu [3]. Vielen aktuellen Beschreibungen und Analysen von Residualzuständen fehlen jedoch die notwendigen Berücksichtigungen bzw. Adjustierungen für den gesundheitlichen Zustand vor der Infektion mit dem Coronavirus. Genau das aber wäre notwendig, um über ein mögliches Post-COVID-Syndrom belastbare Aussagen machen zu können. Jedenfalls wäre es erforderlich, eine klare Abgrenzung zwischen folgenden Umständen vorzunehmen:

1. Symptome bedingt durch eine persistierende chronische Entzündung,
2. Folgen eines Organschadens (akute Lungen- oder Nierenschädigung) und
3. unspezifische Folgen der Hospitalisation und sozialen Isolation (von ernährungsbedingter Anämie bis hin zum Muskelabbau).

Beispielsweise könnten einfache Laboruntersuchungen durchaus beeinflussbare Hintergründe einer Fatigue identifizieren: Anämie, Vitamin-D-Mangel, Hypothyreose, Kortisoldefizit, chronische Nierenerkrankung [4].

## Parallelen zu SARS

Es gibt Erkenntnisse über ein Virus, das dem gegenwärtigen Coronavirus ähnlich ist: SARS‑1, Erreger des schweren akuten respiratorischen Syndroms („severe acute respiratory response syndrome“ [SARS]). Nach der SARS-Pandemie von 2003 fiel auf, dass einige Menschen auch Monate und Jahre nach der Infektion mit dem Virus noch gesundheitliche Probleme hatten: 60 % von 117 Befragten berichteten in einer Studie aus Toronto, wo es den größten Ausbruch außerhalb Asiens gegeben hatte, dass sie noch 1 Jahr nach der Entlassung aus dem Krankenhaus an Fatigue litten [5]. In einer anderen Untersuchung, für die SARS-Überlebende aus Hongkong 4 Jahre nach der Infektion befragt wurden, gaben 40 % an, noch unter Fatigue zu leiden [6]. Und der Schlaf- und Schmerzforscher Harvey Moldofsky identifizierte im Gespräch mit SARS-Überlebenden, die selbst nach einem Reha-Programm nicht wieder arbeiten konnten, eine Reihe von Symptomen: Neben anhaltender Fatigue handelte es sich dabei um diffuse Muskelschmerzen, Schwäche, Depressionen und nicht erholsamen Schlaf. Die Beschwerden fasste er unter dem Namen chronisches Post-SARS-Syndrom zusammen [7].

## Fatigue

Diese Beschwerden ähneln in ihrer Kombination jenen, von denen auch Menschen mit dem chronischen Fatigue-Syndrom (CFS) berichten. Für eine solche Diagnose ist es aber in den meisten Fällen bei COVID-19 jetzt noch zu früh. Dafür müssen die Symptome mindestens 6 Monate anhalten. Aktuell kann man daher wohl nur von postinfektiöser Fatigue sprechen.

Aktuell kann man nur von postinfektiöser Fatigue sprechen

Letztlich ist noch unklar, woher die oftmals geschilderte Abgeschlagenheit kommt. Was aber schon heute sicher ist: Einen einzelnen Grund für die Symptome gibt es nicht. Vielmehr scheinen Veränderungen des Stoffwechsels, des Hormonhaushalts, gegen den eigenen Körper gerichtete Entzündungsbotenstoffe und Veränderungen der Hirnfunktion einen Teil zum Leiden von Fatigue-Patienten beizutragen. Studien deuten etwa darauf hin, dass es den Zellen ihrer Körper schwerer fällt als denen von Gesunden, Energie aus verschiedenen Quellen zu gewinnen [8] – ein wenig so, als würde sich der Körper im Winterschlaf befinden. Eine verminderte Aktivität der Stresshormonachse könnte auch eine gewisse Erschöpfung erklären, denn niedrige Level von Stresshormonen können einerseits dazu führen, dass Entzündungsreaktionen nicht gebremst werden, und andererseits zu einem niedrigen Blutdruck und Kreislaufbeschwerden.

Auch Entzündungsbotenstoffe spielen womöglich eine Rolle. Bei Menschen, die nach einer Virusinfektion eine chronische Fatigue entwickeln, waren in der Akutphase etwa Interleukin‑6 und -10 im Blut stärker erhöht, also exakt die Botenstoffe, die für die überschießende Immunreaktion verantwortlich sind, die COVID-19 so gefährlich macht [9]. Bei postinfektiöser Fatigue sind die entzündlichen Botenstoffe teilweise noch erhöht, obwohl der Mensch schon gesundet ist – im Körper könnte also noch immer eine Entzündung schwelen. Dass eine schwelende Entzündung allein die Fatigue erklären könnte, ist jedoch keinesfalls ausreichend belegt. Es wird gegenwärtig die Hypothese formuliert, proinflammatorische Zytokine (Interferon‑γ, Interleukin-7) könnten in der postinfektiösen Phase die Blut-Hirn-Schranke passieren und autonome Dysfunktionen verursachen, die sich in einer Dysregulation des Schlaf-Wach-Rhythmus, kognitiver Dysfunktion sowie Müdigkeit und Antriebslosigkeit manifestieren können [10].

## Dauerhafte Organschäden und mentale Gesundheitsprobleme

Thromboembolische Komplikationen im Rahmen von COVID-19 wie Pulmonalembolie, Apoplex und andere Mikroinfarzierungen können selbstverständlich eine Vielzahl an dauerhaften Organschäden hervorrufen. Selbst wenn sich Betroffene körperlich wieder erholen, so sind sie evtl. besonders gefährdet, an lang anhaltenden mentalen Gesundheitsproblemen zu leiden. Bei SARS zeigten mehr als ein Drittel der Betroffenen noch 1 Jahr nach körperlicher Erholung eine moderate bis schwere Depression und Ängstlichkeit [11].

### Hyperinflammatorische Zustände

Beobachtungen anderer Rahmenbedingungen, die mit hyperinflammatorischen Zuständen einhergehen, wie SARS, ARDS, Zytokinsturm oder Post-ICU(„intensive care unit“)-Syndrom, erlauben jedenfalls Spekulationen über mögliche Folgen eines kritischen Verlaufes von COVID-19. Eine Hypothese in diesem Zusammenhang ist, dass ein Post-COVID-Syndrom mit einer chronischen subklinischen systemischen Entzündung (Inflammation) einhergehen könnte, wie dies im Alterungsprozess (Aging) beobachtbar ist. Dieses „Inflammaging“ könnte das Potenzial haben, bestehende Komorbiditäten zu verschlechtern und altersabhängige Probleme zu verstärken [12].

Bezüglich der Entzündungsreaktion ist inzwischen bekannt, dass eine SARS-CoV-2-Infektion eine starke und oftmals unkontrollierte Entzündungsantwort auslösen kann, die in der Folge zum Gewebeschaden beitragen kann. Diese als „Zytokinsturm“ bezeichnete schwere, systemische Inflammation wird übrigens in allen Altersgruppen beobachtet, bei Kindern wurde eine schwere Multisysteminflammation mit Ähnlichkeiten zum Kawasaki-Syndrom vielfach beschrieben. Hält eine Entzündungsreaktion jedoch über lange Zeit an, so wird angenommen, dass dies zu zellulärer Seneszenz mit Hemmung der Zellproliferation und Resistenz gegenüber Apoptose führt [12].

### Kognitive, psychologische und physische Einschränkungen

Herridge konnte zeigen, dass Personen nach Erholung von einem ARDS und 5 Jahre nach dem diesbezüglichen Intensivaufenthalt noch immer physische und psychische Limitationen sowie eine reduzierte Lebensqualität empfinden, obwohl sich ihre Lungenfunktionswerte gänzlich oder nahezu vollständig normalisiert hatten [13]. Solche Beobachtungen gibt es jedoch nicht nur nach einem ARDS, viele Intensivpatienten verzeichnen lange anhaltende Beschwerden, die auch als „post-intensive care syndrome“ (PICS) bezeichnet werden. PICS manifestiert sich dabei durch kognitive, psychologische und physische Einschränkungen, die sich in einem relevanten Prozentsatz nicht vollständig zurückbilden [14]. Insofern ist es denkbar, dass eine SARS-CoV-2-Infektion anhaltende Spuren hinterlässt.

### Lungenparenchymveränderungen

Im Rahmen des Ausbruchs von SARS im Jahr 2003 wurden auch längerfristige Auswirkungen wie fibrotische Lungenparenchymveränderungen untersucht. Studienergebnisse zeigten noch 6 Monate nach der Entlassung von Patienten aus der stationären Behandlung eine Einschränkung der Diffusionskapazität bei immerhin 16 % der Betroffenen, und 30 % wiesen zu diesem Zeitpunkt Auffälligkeiten in der Thoraxröntgenaufnahme auf [15]. Bei ARDS (unabhängig von der Ursache) muss bei zumindest 25 % der Überlebenden mit bleibenden Folgen im Sinne einer restriktiven Ventilationsstörung bzw. Lungenerkrankung gerechnet werden [16].

Für die Schwere von bleibenden, in der Bildgebung ersichtlichen bzw. funktionellen Anomalien oder die Wahrscheinlichkeit für Narbengewebe nach ARDS können verschiedene Faktoren eine Rolle spielen. Zu den wichtigsten Einflussfaktoren zählen dabei das Alter des Patienten, vorhandene Komorbiditäten, Raucherstatus, Dauer des Krankenhausaufenthaltes und die Schwere der akuten Erkrankung, beispielsweise die Notwendigkeit einer intensivmedizinischen Versorgung sowie die Art der verabreichten medikamentösen Therapie, also antivirale Medikamente bzw. Kortikosteroide [17].

Radiologisch ersichtliche pulmonale Langzeitfolgen mit Verformung des Lungenparenchyms und konsekutiven Einschränkungen der Lungenfunktion müssen nicht unbedingt der klinischen Symptomatik entsprechen. Zeichen schwerer viraler Pneumonien können in der Bildgebung jedenfalls für einen langen Zeitraum oder sogar dauerhaft ersichtlich bleiben [18]. In diesem Zusammenhang scheint die organisierende Pneumonie in einer signifikanten Anzahl viraler Lungeninfektionen der Ursprung der späteren Veränderungen und Komplikationen zu sein [19]. Bei COVID-19 scheinen die organisierende Pneumonie und der diffuse Alveolarschaden („diffuse alveolar damage“ [DAD]), die sich beide in einer weitgehend vorhersehbaren Art und Weise entwickeln, bei Weitem als häufigste Formen von assoziierten Lungenparenchymschäden aufzutreten [20].

Es bestehen ausreichend Hinweise für die mögliche Existenz eines Post-COVID-Syndroms

In Summe bestehen jedenfalls ausreichend Hinweise für die mögliche Existenz eines „Post-COVID-Syndroms“ bzw. für die Berechtigung, die denkbaren Folgeerscheinungen mit persistierenden Symptomen so zu bezeichnen.

## Rehabilitationsprogramme

Günstig für die vollständige und rasche Genesung sowie die Linderung eines Post-COVID-Syndroms kann sich eine gezielte Rehabilitation auswirken. Solche Rehabilitationsprogramme sind dabei sowohl direkt wie auch auf Distanz („remote rehabilitation“) möglich und effektiv [21]. Obwohl diese Rehabilitationsmaßnahmen im Regelfall einen pneumologischen Schwerpunkt haben, muss gerade auch im Hinblick auf die Vermeidung oder günstige Beeinflussung eines möglichen Post-COVID-Syndroms ebenso auf extrapulmonale (insbesondere neurologische, muskuloskeletale und kardiovaskuläre) Manifestationen und Folgeerscheinungen Bedacht genommen werden [22]. Mit oder ohne erkennbares Post-COVID-Syndrom: Es sind jedenfalls alle Anstrengungen gerechtfertigt, die eine vollständige funktionelle Wiederherstellung und eine Rückkehr in ein Leben nach Corona ermöglichen.

## Fazit für die Praxis

- Bei kritisch kranken COVID-19-Patienten sind körperliche, kognitive und psychologische Folgen der Erkrankung realistisch.
- Für die Diagnose eines Post-COVID-Syndroms ist es in den meisten Fällen bei COVID-19 noch zu früh. Dafür müssen die Symptome mindestens 6 Monate anhalten. Aktuell kann man daher nur von postinfektiöser Fatigue sprechen.
- In Summe bestehen ausreichend Hinweise für die mögliche Existenz eines Post-COVID-Syndroms.
- Um eine vollständige funktionelle Wiederherstellung und eine Rückkehr in ein Leben nach Corona zu ermöglichen, sollten gezielte Rehabilitationsmaßnahmen in Erwägung gezogen werden.
